# A case of transanal protrusion of ventriculoperitoneal shunt in an adult patient without any classic symptoms of bowel perforation

**DOI:** 10.1002/ccr3.8983

**Published:** 2024-05-26

**Authors:** Kimia Mirjalali, Sarah Seyedyousefi

**Affiliations:** ^1^ Department of Surgery Isfahan University of Medical Sciences Isfahan Iran

**Keywords:** anal protrusion, bowel perforation, hydrocephalus, traumatic brain injury, ventriculoperitoneal shunts

## Abstract

**Key Clinical Message:**

This report emphasizes the significance of acknowledging infrequent yet severe complications such as bowel perforation and transanal protrusion post ventriculoperitoneal shunt (VPS) surgery. VPS patients should be observed for atypical indicators and manifestations that could suggest the presence of such complications, even in the lack of traditional clinical signs of peritonitis or bowel perforation.

**Abstract:**

Placing an intracranial shunt, may be a reasonable approach to decrease the complications of hydrocephalus and it can be done either simultaneous to cranioplasty or not. Ventriculoperitoneal shunts were first proposed in 1905 and has been used since. Similar to any other procedure, there are different complications to this surgery. Abdominal complications, including peritoneal pseudocysts, intestinal volvulus, protruding in hernial sac or extrusion through vagina, scrotum, umbilicus or gastrointestinal tract, are rare but according to previous studies happen in 5%–47% of cases. Bowel perforation is a rare complication and can happen in 0.01%–0.07% of patients. It's also worth mentioning that only 25% of patients with bowel perforation experience the classic clinical symptoms of peritonitis or bowel perforation. This particular complication should not be overlooked since it can cause a high mortality rate of 15%. Here we present a case of transanal protrusion of ventriculoperitoneal shunt after an asymptomatic bowel perforation, in an adult who has undergone surgery after a traumatic brain injury. The patient has undergone surgery and lastly the shunt was manually removed from anus. He was monitored for 3 days and eventually discharged.

## 1 INTRODUCTION

Hydrocephalus, either congenital or acquired, has long been known to surgeons and many different treatment approaches have been proposed.^1^ Hydrocephalus happening after a traumatic brain injury has also been studied in multiple studies. Traumatic brain injury is a leading cause of death worldwide, especially in low‐ and middle‐income countries and its prevalence is considerable.^2^ One of the popular ways of treatment to decrease intracranial pressure after a brain injury or hemorrhage, is a surgery known as decompressing craniotomy (DC) which may be a major risk factor in occurrence of post‐traumatic hydrocephalus.^2^

Placing an intracranial shunt, may be a reasonable approach to decrease the complications of hydrocephalus and it can be done either simultaneous to cranioplasty or not.^2^ Ventriculoperitoneal shunts were first proposed by Kausch in 1905 and has been used since.^1^ Similar to any other procedure, there are different complications to this surgery. Abdominal complications, including peritoneal pseudocysts, intestinal volvulus, and protruding in hernial sac or extrusion through vagina, scrotum, and umbilicus or gastrointestinal tract, are rare but according to previous studies happen in 5%–47% of cases.^3^, ^4^, ^5^

Bowel perforation is a rare complication and can happen in 0.01%–0.07% of patients. It is also worth mentioning that only 25% of patients with bowel perforation experience the classic clinical symptoms of peritonitis or bowel perforation. This particular complication should not be overlooked since it can cause a high mortality rate of 15%.^4^, ^6^, ^7^

There has been other case reports on this subject, presenting cases suffering bowel perforation and anal extrusion of the ventriculoperitoneal shunt (VPS).^8^, ^9^ These cases have been mostly reported in children.

Here we present a case of transanal protrusion of vps shunt, in an adult who has undergone surgery after a traumatic brain injury.

## 2 CASE PRESENTATION

We present the case of a 36‐year‐old male who suffered multiple trauma after a falling from 4 m height. The patient had traumatic brain injury and the CT scan showed a subdural hematoma in both hemispheres. Thus, the patient underwent decompressing craniotomy.

After a month, the patient developed post‐traumatic hydrocephalus and a VP shunt was put into left lateral ventricle and a cranioplasty was done simultaneously.

The patient was followed and 6 months later, the VPS placement surgery was redone after a shunt malfunction was diagnosed.

A month later, the patient showed up with a history of worsening headaches and was scheduled for surgery with a diagnosis of recurrent shunt malfunction. The proximal part of the VPS was removed from the left ventricle but the distal part could not be removed despite trying and eventually was left in place. Afterwards, another VP Shunt was placed into right lateral ventricle.

**FIGURE 1:**
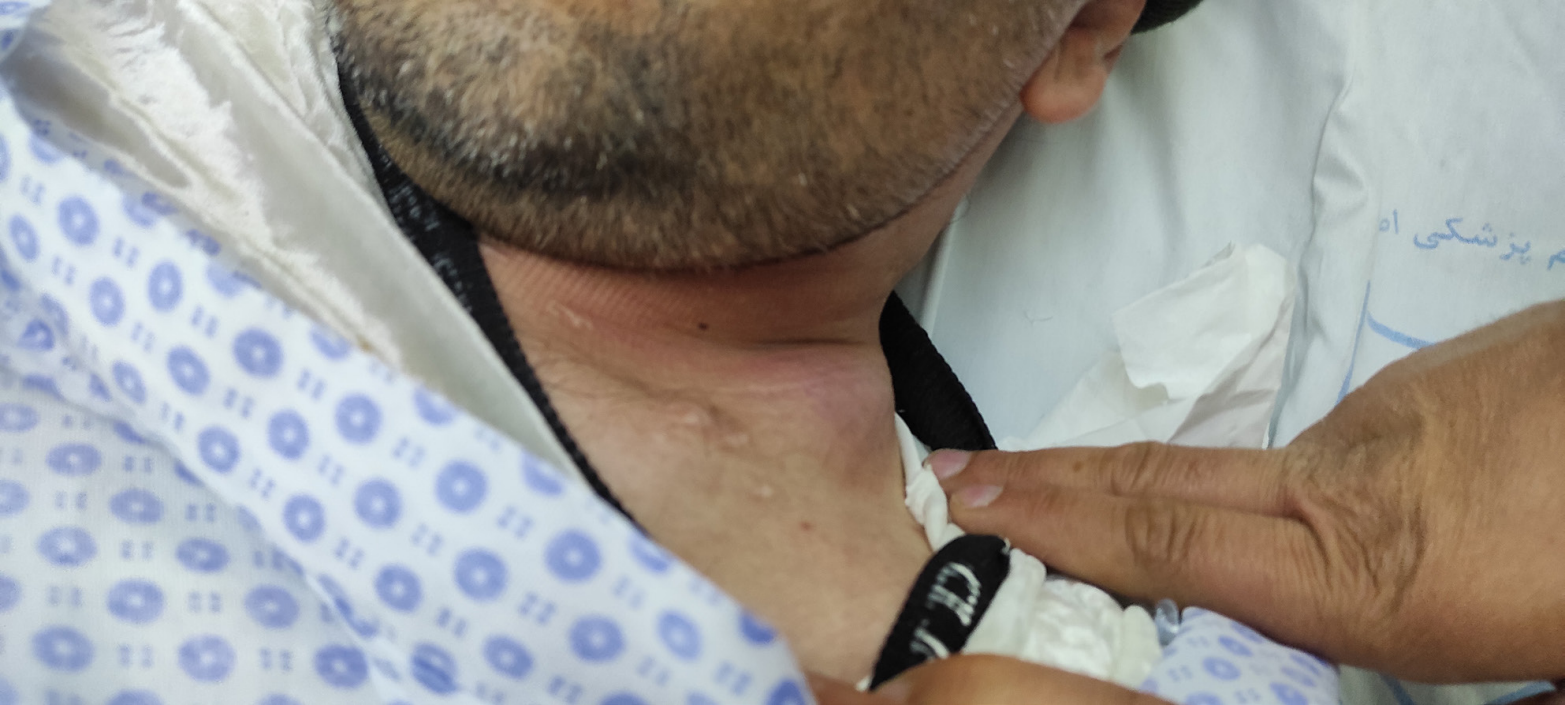
Supraclavicular bulge containing air.

About a year later, the patient showed up to ER with complaint of a swelling sized 3 × 4 cm in left supraclavicular region (Figure 1).

## 3 METHODS

**FIGURE 2:**
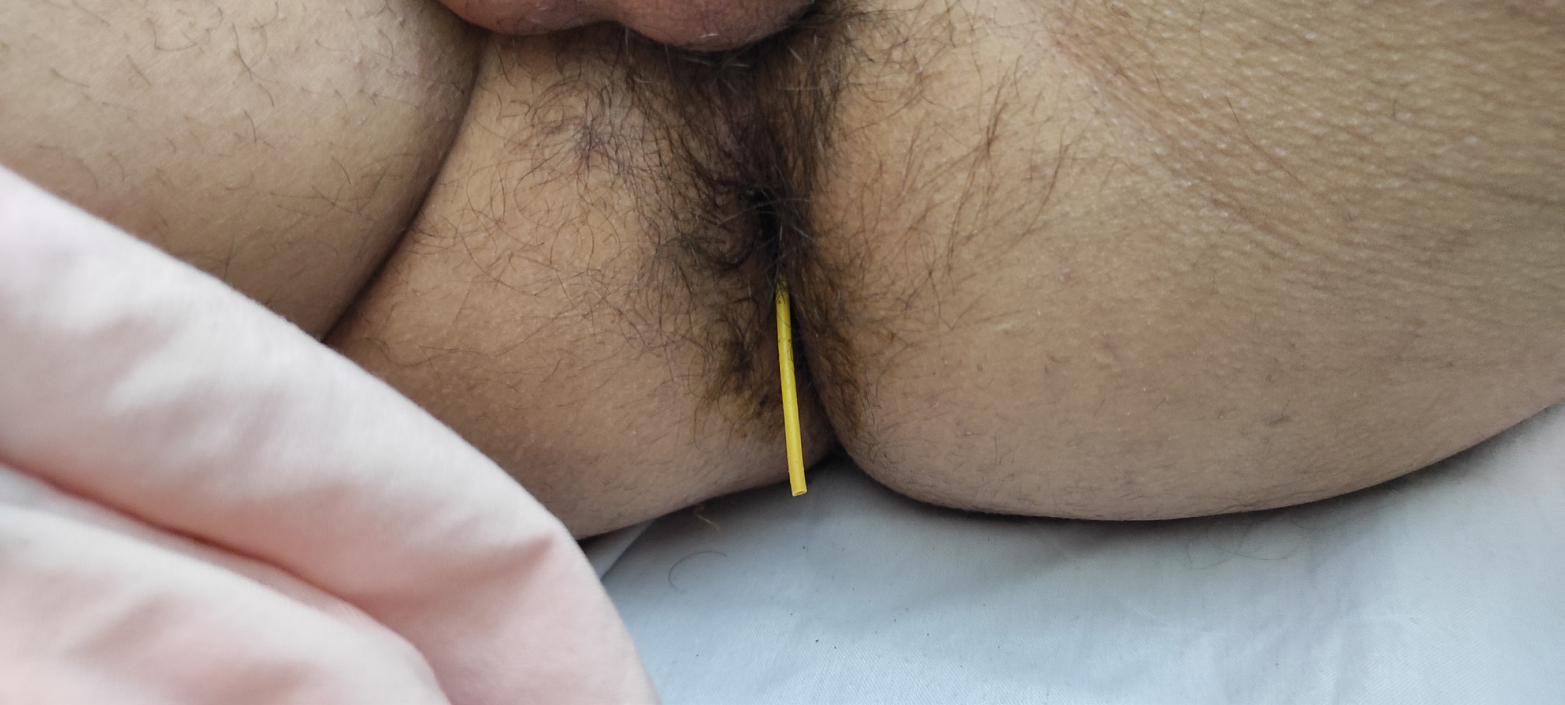
Trans‐anal protrusion of the shunt.

Knowing about the patient previous history and the history of the VPS, a pus collection was suspected. The patient had no other symptoms and no history of fever or pain. The patient was scheduled for an ultrasound study and surprisingly a collection of air was confirmed. The patient was thoroughly examined and it was observed that a yellowish tube was protruding from the anus (Figure 2).

**FIGURE 3:**
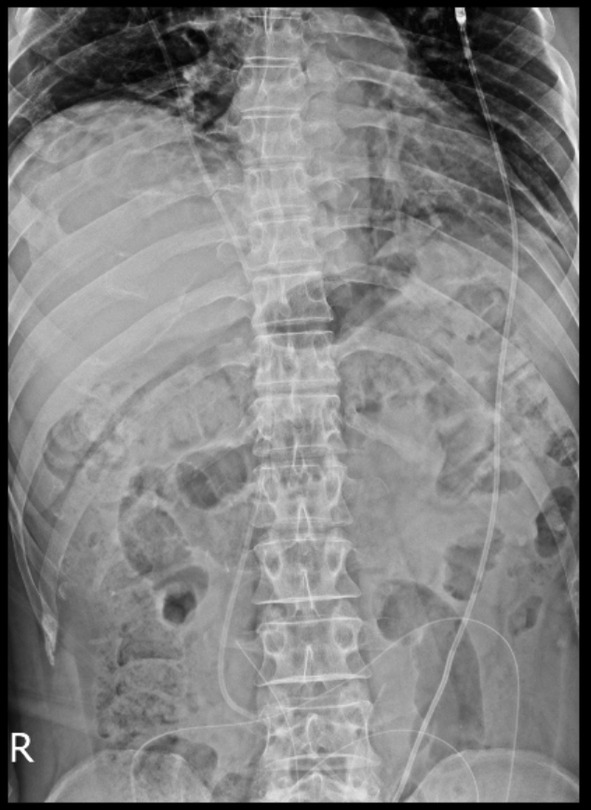
Abdominal x‐ray showing the proximal part of the shunt.

**FIGURE 4:**
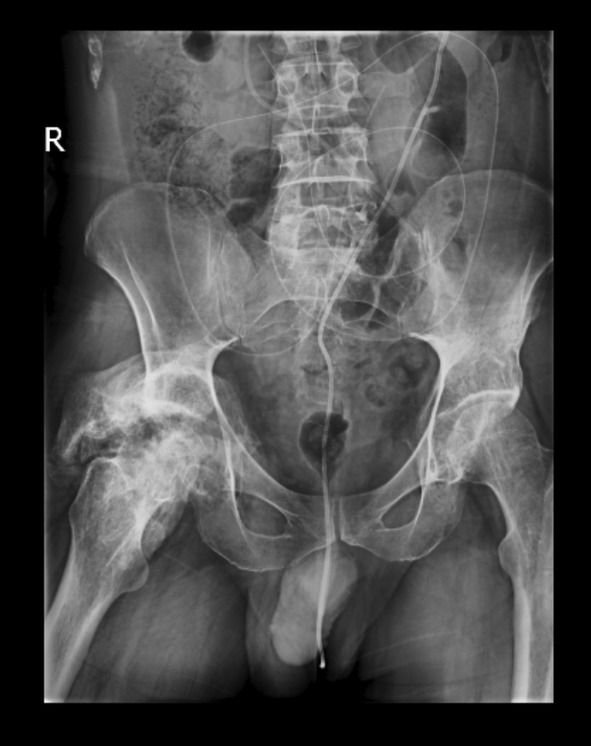
Abdominopelvic x‐ray showing both shunts and the protrusion.

The air collection was aspirated and, in the end, the remaining air was absorbed on its own. The patient was scheduled for x‐ray radiology for abdominal and chest x‐rays. And the shunt was seen in the radiographies, protruding through the rectum and the anus. Radiology images are shown in Figures 3 and 4.

Knowing patient's previous history, an asymptomatic intestinal or colic perforation or an anal fistula due to displacement and migration of ventriculoperitoneal shunt was suspected. We suspected that the shunt has perforated the colon, entered the colon and the rectum and eventually protruded through anus. Fortunately, like most cases, our case was asymptomatic and had no symptoms of peritonitis or other complications. After a consult with the neurosurgery team of the hospital, the patient was prepped for surgery.

The shunt's proximal part was located using a C‐Arm imaging and the location was marked on the patient's chest to be in the third costal space. Using a transverse incision, the proximal and metal part of the shunt (about 2–3 cm) was cut, separated and taken out. After taking out the proximal part, the distal part was removed from anus.

## 4 RESULTS AND CONCLUSION

Due to a suspicion of perforation, the patient was monitored for 3 days in the hospital and did not develop any symptom indicating bowel perforation or peritonitis. It is worth mentioning that the patient did not have any symptoms indicating any gastrointestinal issues. Considering the chronic occurrence of the complication, the patient being asymptomatic, low economic state of the patient and no problems happening during surgery and removal of shunts, colonoscopy did not seem necessary.^8^

After antibiotic therapy and monitoring, the patient was referred to neurosurgeons to make an appointment for following up the post‐traumatic hydrocephalus and shunt function.

A month later, the patient was followed again and had no gastrointestinal discomfort or symptoms.

## 5 DISCUSSION

Using shunts and diversion of cerebrospinal fluid through them has long been used in hydrocephalus, either congenital acquired or post‐traumatic hydrocephalus. Ventriculoperitonal shunts, was first proposed by Kausch and has been used since as an effective treatment approach.^1^, ^10^

Like any other procedure, there are different complications to this surgery. Abdominal complications, including peritoneal pseudocysts, intestinal volvulus, protruding in hernial sac or extrusion through vagina, scrotum, umbilicus, or gastrointestinal tract, are rare but according to previous studies can happen in 5%–47% of cases.^3^, ^4^, ^5^ A list of reported cases in previous literature has been shown in the Table S1.

Bowel perforation is a rare complication and can happen in 0.01%–0.07% of patients. Seventy‐five percent of patients with bowel perforation do nott experience the classic clinical symptoms of peritonitis or bowel perforation and may be asymptomatic. This particular complication should not be overlooked since it can cause a high mortality rate of 15% and although rare, may cause severe consequences.^4^, ^5^, ^7^

VP shunt anal protrusion is an extremely rare complication which have been reported less than a 100 times in literature. Most of the cases reported with this complication, suffered this condition in months after surgery and most of them happened in children and were asymptomatic.^8^, ^9^, ^10^, ^11^

In these cases, different aspects should be considered carefully. One of the most important parts of the management aside from the removal of the case, is a complete work‐up on CSF (Cerebrospinal fluid) contamination, meningitis, ventriculitis, sepsis, perforative peritonitis, and peritoneal abscess formations. Each of these occurrences may have an important impact on the course of treatment and may increase the mortality rate up to 15%.^8^

In cases of intestinal perforation without any other complications, different approaches have been suggested. It has been recommended that in the acute cases of perforation with gastrointestinal symptoms, or signs of peritonitis, an emergency laparotomy should be done in order to remove the shunt or repair the perforation; in additional, a peritoneal lavage may be indicated.^8^, ^9^, ^12^ Removing the distal end of the shunt, either manually through the anus or during laparotomy, should be done with extreme caution in order to minimize the probable contamination of the peritoneal cavity and CSF. In the previous literature, laparoscopic management of this situation and removal of the shunt have also been suggested.^8^, ^13^

The patient should receive broad‐spectrum antibiotics for at least 3 weeks. In order to do a full work‐up on CSF, multiple CSF cultures must be sent and after negative results are verified, the patient can be observed and prepped for another shunt placement surgery, either on the other side of the brain or on the same side.^9^

Some measures need to be taken in order to prevent this complication. Anchoring the shunt to the intestinal wall or peritoneum can decrease the chance of perforation presumably.^10^, ^14^ But no study with a long‐term follow‐up has been conducted in this regard and this could be a study gap to be explored in the future.

Here, we presented a case of trans‐anal protrusion of VP shunt in a 36‐year‐old man of post‐traumatic hydrocephalus, 1 year after the shunt placement. The patient underwent surgery and the shunt was successfully removed manually through the anus. Like many other cases, there were no peritoneal or gastrointestinal symptoms in our patient and fortunately, the shunt had no connection to the ventricle when this complication happened; therefore, the chances of CSF contamination were minimal. The patient was monitored for 3 days, receiving broad‐spectrum antibiotics and was then referred to neurosurgeons for subsequent measures.

## 6 CONCLUSION

Although bowel perforation and trans‐anal protrusion of the ventriculoperitoneal shunt is an extremely rare complication of VP shunt placement, it can lead to serious features such as ascending gram‐negative meningitis, ventriculitis, sepsis, perforative peritonitis, and peritoneal abscess. Hence knowing this complication and having it in mind when treating patients with a ventriculoperitoneal shunt might be crucial for both general surgeons and neurosurgeons. Obviously, as more reports and management approaches get published, better treatments or even guidelines might get designed.

## AUTHOR CONTRIBUTIONS

**Kimia Mirjalali:** Investigation; project administration; writing – review and editing. **Sarah Seyedyousefi:** Investigation; writing – original draft; writing – review and editing.

## FUNDING INFORMATION

The others received no external funding from any institutions.

## CONFLICT OF INTEREST STATEMENT

The authors declare no conflict of interest considering this publication.

## CONSENT

Written informed consent was obtained from the patient to publish this report in accordance with the journal's patient consent policy.

## Supporting information


Table S1.

## Data Availability

Data is available upon request from the corresponding author.
