# Supplementary material for: A case of transanal protrusion of ventriculoperitoneal shunt in an adult patient without any classic symptoms of bowel perforation
Source: Clin Case Rep. 2024 May 26;12(6):e8983. doi: 10.1002/ccr3.8983 (PMC11128491; doi:10.1002/ccr3.8983)
Supplement: Supplementary file 1 — Table S1. [file CCR3-12-e8983-s001.docx]

| Authors | Year | Number of cases | Complication | References |
| --- | --- | --- | --- | --- |
| Karshe et al | 2024 | 1 | Bowel Perforation and transanal extrusion | (1) |
| Awwad et al | 2024 | 1 | Bowel Perforation | (2) |
| Rinad AlJoaid et al | 2023 | 1 | Bowel perforation and obstruction | (3) |
| Morinaga et al | 2023 | 1 | Bowel perforation and peritonitis | (4) |
| Fernandez et al | 2022 | 1 | Bowel perforation | (5) |
| Scarascia et al | 2022 | 1 | Gastric perforation | (6) |
| Nakamura et al | 2022 | 1 | Bowel perforation | (7) |
| Bosy et al | 2021 | 1 | Bowel perforation and anal extrusion | (8) |
| Van Dong et al | 2021 | 1 | Bowel perforation | (9) |
| Pant et al | 2021 | 5 | Two cases of intestinal obstruction secondary to small bowel perforation /1 case of with gastric perforation and per-oral presentation of the shunt, 2 cases rectal perforation with per-anum extrusion of the shunt | (10) |
| Prabhat et al | 2020 | 1 | Bowel perforation and enterocutaneous fistula | (11) |
| Chen et al | 2020 | 1 | Bowel perforation | (12) |
| Bakshi et al | 2020 | 1 | Bowel perforation and anal extrusion | (13) |
| Marino et al | 2019 | 1 | Bowel perforation and anal extrusion | (14) |
| Ezzat et al | 2018 | 8 | 6 cases of anal extrusion  1 case of oral extrusion  1 case of colonic perforation | (15) |
| Cohen-Addad | 2018 | 1 | Gastric perforation | (16) |
| Czajka et al | 2018 | 1 | Cecal perforation | (17) |
| Badri et al | 2018 | 1 | Transoral migration | (18) |
| Hasan et al | 2018 | 2 | Anal extrusion | (19) |
| Osman et al | 2017 | 1 | Bowel perforation | (20) |
| Al Fauza et al | 2017 | 1 | Oral extrusion | (21) |
| Ricardello et al | 2016 | 1 | Bowel Perforation | (22) |
| Bourm et al | 2016 | 1 | Bowel perforation | (23) |
| Thiong’o et al | 2015 | 6 | 6 children were identified whose VP shunts had perforated the GI tract; 2 shunts subsequently protruded through the anal orifice, 1 protruded through the oral cavity | (24) |
| Bansal et al | 2015 | 1 | Bowel perforation and anal extrusion | (25) |
| Voronovich et al | 2014 | 1 | Bowel perforation and enterocutaneous fistula | (26) |
| Knuth et al | 2013 | 1 | Bowel perforation | (27) |
| Rinker et al | 2013 | 1 | Bowel perforation | (28) |
| Sharifian et al | 2013 | 1 | Bowel Perforation and transanal extrusion | (29) |
| Yilmaz et al | 2013 | 1 | Gastric perforation and transoral extrusion | (30) |
| Ozturk et al | 2012 | 1 | Bowel Perforation and transanal extrusion | (31) |
| Mihajlovic et al | 2012 | 1 | Bowel perforation | (32) |
| Hayama et al | 2011 | 1 | Bowel Perforation and transanal extrusion | (33) |
| Hai et al | 2011 | 2 | Bowel perforation | (34) |
| Low et al | 2010 | 1 | Bowel perforation and Oral extrusion | (35) |
| Chiang et al | 2010 | 1 | Bowel perforation | (36) |
| Shaw et al | 2008 | 1 | Bowel perforation | (37) |
| Matsuoka et al | 2008 | 1 | Bowel Perforation and transanal extrusion | (38) |
| Berhouma et al | 2008 | 1 | Bowel perforation and transoral extrusion | (39) |
| Ghritlahrey et al | 2007 | 10 | Bowel Perforation and transanal extrusion | (40) |
| Hernandez-magro et al | 2006 | 1 | Bowel perforation | (41) |
| Guillen et al | 2002 | 1 | Bowel perforation | (42) |
| Chen et al | 2000 | 1 | Bowel perforation | (43) |
| Digray et al | 2000 | 1 | Bowel perforation and transanal extrusion | (44) |
| Ibrahim et al | 1998 | 1 | Bowel perforation and transanal extrusion | (45) |
| Karibe et al | 1998 | 1 | Bowel perforation | (46) |
| Panagea et al | 1997 | 2 | Bowel perforation | (47) |
| Kin et al | 1997 | 1 | Bowel perforation | (48) |
| Alonso Vanegas et al | 1994 | 1 | Gastric Perforation | (49) |
| Jamjoom et al | 1990 | 1 | Bowel perforation | (50) |
| Ogawasara et al | 1990 | 1 | Bowel perforation | (51) |
| Abu-Dalu et al | 1983 | 1 | Bowel Perforation | (52) |
| Agha et al | 1983 | 1 | Bowel Perforation | (53) |
| Nishijima et al | 1980 | 1 | Gastric Perforation | (54) |

Supplementary table 1 – a list of previous reports regarding immigration of VPS and gastric or Bowel perforations

1. Karshe NA, Osman FAO, Elmi AM, Mohamed Dirie A. Anal extrusion of silent migrated ventriculoperitoneal shunt: Case report and literature review. Radiol Case Rep. 2024;19(4):1524-7.

2. Awwad AR, M SHO, Asad D, B YMHAR, A MMD, S NMB, et al. Ventriculoperitoneal Shunt Migration Into the Transverse Colon: A Case Report. Cureus. 2024;16(1):e52334.

3. AlJoaid RM, Alshakhori HH, Haji A, Alfaraj D, Alabbad MF. Closed-Loop Bowel Obstruction Induced by Ventriculoperitoneal Shunt Catheter Coiling at the Sigmoid Colon: A Case Report. Cureus. 2023;15(11):e49045.

4. Morinaga T, Nakahara O, Tsuji A, Kuramoto K, Iizaka M, Hayashida S, et al. Laparoscopic intervention for late-onset perforating peritonitis due to a ventriculoperitoneal shunt: a case report and literature review. Surg Case Rep. 2023;9(1):154.

5. Fernandez B, Gautier A, Koumaré IB, Fabre JM, Coubes P, Poulen G. Transcutaneous ventriculo-peritoneal shunt catheter extrusion with silent bowel perforation following digestive surgery: a case report. Br J Neurosurg. 2022:1-4.

6. Scarascia A, Atallah E, Pineda MA, Rosenwasser R, Judy K. Gastric perforation from a migrating ventriculoperitoneal shunt: A case report and review of literature. Radiol Case Rep. 2022;17(12):4899-902.

7. Nakamura H. [A case of bacterial meningitis due to colon perforation of V-P shunt tube in an elderly patient with prolonged high fever after hospitalization for urinary tract infection]. Nihon Ronen Igakkai Zasshi. 2022;59(4):551-8.

8. Bosy HH, Albarnawi BM, Ashour KM, Alyasi A, Alsulaihebi AS. Early Anal Protrusion of Distal Ventriculoperitoneal Catheter Due to Iatrogenic Colonic Perforation: A Case Report and Review of Literature. Cureus. 2021;13(12):e20296.

9. Van Dong H, Van HD, Vu HT, Chu HT. Duodenal perforation as a postoperative complication after ventriculoperitoneal shunt: A case report. Int J Surg Case Rep. 2021;83:106059.

10. Pant N, Singh S, Singh G, Kumar A, Rai RK, Rawat J, et al. The wandering ventriculoperitoneal shunt and the scope of its salvage. Childs Nerv Syst. 2021;37(8):2613-8.

11. Prabhat J, Dhoj JB. Case report of enterocutaneous fistula due to non-functioning ventriculoperitoneal shunt. J Surg Case Rep. 2020;2020(9):rjaa378.

12. Chen YH, Hsieh CT, Sun JM, Chang SI. Gastric perforation by a ventriculoperitoneal shunt in an adult. Neurosciences (Riyadh). 2020;25(2):144-7.

13. Bakshi S. Spontaneous trans-anal extrusion of caudally migrated ventriculo-peritoneal shunt tip in a child: a case report. Surg Case Rep. 2020;6(1):50.

14. Marino M, Phillips C. Methicillin-Resistant Staphylococcus aureus Meningitis from Transanal Migration of a Ventriculoperitoneal Shunt. J Emerg Med. 2019;57(3):e81-e4.

15. Ezzat AAM, Soliman MAR, Hasanain AA, Thabit MA, Elshitany H, Kandel H, et al. Migration of the Distal Catheter of Ventriculoperitoneal Shunts in Pediatric Age Group: Case Series. World Neurosurg. 2018;119:e131-e7.

16. Cohen-Addad DI, Hewitt K, Bell D. A ventriculoperitoneal shunt incidentally found in the stomach. Radiol Case Rep. 2018;13(6):1159-62.

17. Czajka G, Kreminski H. Cecal perforation: A rare complication of a ventriculoperitoneal shunt. Jaapa. 2018;31(9):28-31.

18. Badri M, Gader G, Belkahla G, Kallel J, Zammel I. Transoral migration of the inferior end of a ventriculoperitoneal shunt: A case report with literature review. Neurochirurgie. 2018;64(3):203-5.

19. Hasan A, Sharma S, Chopra S, Purohit DK. Anal Extrusion of Ventriculoperitoneal Shunt: A Report of Two Cases and Review of Literature. J Pediatr Neurosci. 2018;13(1):8-12.

20. Osman B, Roushias S, Hargest R, Narahari K. Migration of ventriculoperitoneal shunt to urethral and rectal orifices. BMJ Case Rep. 2017;2017.

21. Al Fauzi A, Suryaningtyas W, Wahyuhadi J, Parenrengi MA, Turchan A, Wijaya MC, et al. Upward migration and peroral extrusion of a peritoneal shunt catheter: Case report and review of the literature. Surg Neurol Int. 2017;8:178.

22. Riccardello GJ, Jr., Barr LK, Bassani L. Bowel perforation presenting with acute abdominal pain and subcutaneous emphysema in a 14-year-old girl with an abandoned distal peritoneal shunt catheter: case report. J Neurosurg Pediatr. 2016;18(3):325-8.

23. Bourm K, Pfeifer C, Zarchan A. Small bowel perforation: a rare complication of ventriculoperitoneal shunt placement. J Radiol Case Rep. 2016;10(6):30-5.

24. Thiong'o GM, Luzzio C, Albright AL. Ventriculoperitoneal shunt perforations of the gastrointestinal tract. J Neurosurg Pediatr. 2015;16(1):36-41.

25. Bansal H, Gupta G, Gupta M, Kaushal R. Unusual Ventriculoperitoneal (VP) Shunt Tube Extrusion through Anus in a Child with Dandy Walker Malformation: A Rare Case Report. J Clin Diagn Res. 2015;9(1):Pd25-6.

26. Voronovich ZA, Albright AL. Enterocutaneous fistula in the setting of ventriculoperitoneal shunt extrusion through the skin and perforation through the small bowel. J Neurosurg Pediatr. 2014;14(4):340-3.

27. Knuth J, Detzner M, Heiss MM, Weber F, Bulian DR. Laparoscopy for a ventriculoperitoneal shunt tube dislocated into the colon. Jsls. 2013;17(4):675-8.

28. Rinker EK, Osborn DA, Williams TR, Spizarny DL. Asymptomatic bowel perforation by abandoned ventriculoperitoneal shunt. J Radiol Case Rep. 2013;7(9):1-8.

29. Sharifian A, Abdollahi A, Maddah G, Anaraki F, Alvandipour M, Abbasi Sahebi M, et al. Spontaneous transanal protrusion of ventriculoperitoneal catheter: a case report. Acta Med Iran. 2013;51(2):135-8.

30. Yilmaz MB, Egemen E, Tonge M, Kaymaz M. Transoral protrusion of a peritoneal catheter due to gastric perforation 10 years after a ventriculoperitoneal shunting: case report and review of the literature. Turk Neurosurg. 2013;23(2):285-8.

31. Ozturk H, Is M, Ozturk H, Kucuk A, Dosoglu M. Transanal protrusion of a ventriculoperitoneal shunt catheter. J Coll Physicians Surg Pak. 2012;22(11):733-4.

32. Mihajlović M, Tasić G, Raicević M, Mrdak M, Petrović B, Radlović V. Asymptomatic perforation of large bowel and urinary bladder as a complication of ventriculoperitoneal shunt: report of two cases. Srp Arh Celok Lek. 2012;140(3-4):211-5.

33. Hayama T, Ishihara S, Yamazaki N, Akahane T, Shimada R, Horiuchi A, et al. Severance of a ventriculoperitoneal shunt catheter implanted between the cerebral ventricle and peritoneal cavity, resulting in protrusion from the anus. Int Surg. 2011;96(2):148-52.

34. Hai A, Rab AZ, Ghani I, Huda MF, Quadir AQ. Perforation into gut by ventriculoperitoneal shunts: A report of two cases and review of the literature. J Indian Assoc Pediatr Surg. 2011;16(1):31-3.

35. Low SW, Sein L, Yeo TT, Chou N. Migration of the abdominal catheter of a ventriculoperitoneal shunt into the mouth: a rare presentation. Malays J Med Sci. 2010;17(3):64-7.

36. Chiang LL, Kuo MF, Fan PC, Hsu WM. Transanal repair of colonic perforation due to ventriculoperitoneal shunt--case report and review of the literature. J Formos Med Assoc. 2010;109(6):472-5.

37. Shaw A, Lund JN, Semeraro D, Cartmill M, Reynolds JR, Tierney GM. Large bowel obstruction and perforation secondary to endometriosis complicated by a ventriculoperitoneal shunt. Colorectal Dis. 2008;10(5):520-1.

38. Matsuoka H, Takegami T, Maruyama D, Hamasaki T, Kakita K, Mineura K. Transanal prolapse of a ventriculoperitoneal shunt catheter--case report. Neurol Med Chir (Tokyo). 2008;48(11):526-8.

39. Berhouma M, Messerer M, Houissa S, Khaldi M. Transoral protrusion of a peritoneal catheter: a rare complication of ventriculoperitoneal shunt. Pediatr Neurosurg. 2008;44(2):169-71.

40. Ghritlaharey RK, Budhwani KS, Shrivastava DK, Gupta G, Kushwaha AS, Chanchlani R, et al. Trans-anal protrusion of ventriculo-peritoneal shunt catheter with silent bowel perforation: report of ten cases in children. Pediatr Surg Int. 2007;23(6):575-80.

41. Martinez Hernández-Magro P, Barrera Román C, Villanueva Sáenz E, Zavala MJ. Colonic perforation as a complication of ventriculoperitoneal shunt: a case report. Tech Coloproctol. 2006;10(4):353-5.

42. Guillén A, Costa JM, Castelló I, Claramunt E, Cardona E. [Unusual abdominal complication of ventriculoperitoneal shunt]. Neurocirugia (Astur). 2002;13(5):401-4.

43. Chen HS. Rectal penetration by a disconnected ventriculoperitoneal shunt tube: an unusual complication. Chang Gung Med J. 2000;23(3):180-4.

44. Digray NC, Thappa DR, Arora M, Mengi Y, Goswamy HL. Silent bowel perforation and transanal prolapse of a ventriculoperitoneal shunt. Pediatr Surg Int. 2000;16(1-2):94-5.

45. Ibrahim AW. E. coli meningitis as an indicator of intestinal perforation by V-P shunt tube. Neurosurg Rev. 1998;21(2-3):194-7.

46. Karibe H, Ishibashi Y. [A case of sigmoid colon perforation by a V-P shunt tube resulting in pneumocephalus]. No Shinkei Geka. 1998;26(1):79-82.

47. Panagea S, Cartmill TD, Panigrahi H. Intracerebral sepsis due to intestinal perforation by ventriculo-peritoneal shunts: two cases. J Infect. 1997;35(1):86-8.

48. Kin S, Imamura J, Ikeyama Y, Jimi Y, Yasuhara S. [Perforation of the intestine by a peritoneal tube 10 years after a ventriculo-peritoneal shunt]. No Shinkei Geka. 1997;25(6):573-5.

49. Alonso-Vanegas M, Alvarez JL, Delgado L, Mendizabal R, Jiménez JL, Sanchez-Cabrera JM. Gastric perforation due to ventriculo-peritoneal shunt. Pediatr Neurosurg. 1994;21(3):192-4.

50. Jamjoom AB, Rawlinson JN, Kirkpatrick JN. Passage of tube per rectum: an unusual complication of a ventriculoperitoneal shunt. Br J Clin Pract. 1990;44(11):525-6.

51. Ogasawara K, Kaneko U, Ishibashi T, Sakimura K. [Abscess of the abdominal wall following penetration of the descending colon: a rare complication of a ventriculoperitoneal shunt]. No Shinkei Geka. 1990;18(10):975-8.

52. Abu-Dalu K, Pode D, Hadani M, Sahar A. Colonic complications of ventriculoperitoneal shunts. Neurosurgery. 1983;13(2):167-9.

53. Agha FP, Amendola MA, Shirazi KK, Amendola BE, Chandler WF. Unusual abdominal complications of ventriculo-peritoneal shunts. Radiology. 1983;146(2):323-6.

54. Nishijima M, Ohyama H, Higuchi H. [Gastric perforation in ventriculo-peritoneal shunt--a case report (author's transl)]. No Shinkei Geka. 1980;8(7):679-83.
